# Response to COVID-19 recommended preventive behavioral messages among Guraghe zone communities, South Ethiopia: using constructs of the Extended Parallel Process Model (EPPM)

**DOI:** 10.1186/s12879-023-08087-1

**Published:** 2023-03-28

**Authors:** Abdurezak Kemal, Kenzudin Assfa, Bisrat Zeleke, Mohammed Jemal, Musa Jemal, Shemsu Kedir, Amare Zewdie, Samuel Dessu, Fedila Yassin, Adane Habtie

**Affiliations:** 1 Department of Public Health, College of Health Sciences and Medicine, Wolkite University, Wolkite, Ethiopia; 2 Department of Health, Behavior, and Society, Faculty of Public Health, Institute of Health, Jimma University, Jimma, Ethiopia; 3 Department of Public Health, College of Medicine and Health Sciences, Werabe University, Werabe, Ethiopia

**Keywords:** COVID-19, Gurage zone, Extended parallel process model

## Abstract

**Introduction:**

The World Health Organization declared COVID-19 is a pandemic disease. Countries should take standard measures and responses to battle the effects of the viruses. However, little is known in Ethiopia regarding the recommended preventive behavioral messages responses. Therefore, the study aimed to assess the response to COVID-19 recommended preventive behavioral messages.

**Methods:**

Community-based cross-sectional study design was carried out from 1 to 20, July 2020. We recruited 634 respondents by using a systematic sampling method. Data were analyzed using Statistical Package Software for Social Sciences version 23. Association between variables were explored using a bivariable and multi variable logistic regression model. The strength of the association is presented using odds ratio and regression coefficient with 95% confidence interval. A p-value of less than 0.05 was declared statistically significant.

**Results:**

Three hundred thirty-six (53.1%) of respondents had good response to recommended preventive behavioral messages. The general precise rate of the knowledge questionnaire was 92.21%. The study showed that merchant was 1.86 (p ≈ 0.01) times more likely respond to COVID-19 recommended preventive behavioral messages than government-employed. Respondents who scored one unit increase for self-efficacy and response-efficacy, the odds of responding to COVID-19 recommended preventive behavioral messages were increased by 1.22 (p < 0.001), and 1.05 times (p = 0.002) respectively. Respondents who scored one unit increase to cues to action, the odds of responding to COVID-19 recommended preventive behavioral messages were 43% (p < 0.001) less likely.

**Conclusion:**

Even though respondents were highly knowledgeable about COVID-19, there is a lower level of applying response to recommended preventive behavioral messages. Merchant, self-efficacy, response efficacy, and cues to action were significantly associated with response to recommended preventive behavioral messages. Like merchants, government employer should be applying preventive behavioral messages and also, participants’ self and response efficacy should be strengthened to improve the response. In addition, we should be changed or modified the way how-to deliver relevant information, promoting awareness, and also using appropriate reminder systems to preventive behavioral messages.

## Introduction

The World Health Organization declared COVID-19 is a pandemic disease [1]. Countries should take standard measures and responses to tackle the effects of the viruses. The global community is struggling to decrease and finally halt the spread of Covid-19 through improving the knowledge and practice of COVID-19 prevention methods, testing, and screening [2, 3].

Among the general public, we focus on practicing protective behaviors which intended for behavioral response. To oblige human-to-human transmission, public health interventions, such as hand hygiene and wearing masks, are performed [4, 5]. To prevent transmission of the virus during infectious disease pandemics following proper handwashing hygiene is an activity that is often recommended by health authorities [6], preventive behaviors that was frequently researched [7], and also have confirmed to affect the feast of pandemics [8] significantly. Furthermore, the protective effect of wearing facial masks to reduce respiratory virus transmission is widely supported in the literature [9, 10].

The Korean government were recommended practicing hand hygiene and wearing facial masks. During pandemic frequently occur avoidance behaviors due to fear of transmission were canceling or postponing social events, reducing the use of public transport, keeping children out of school, and avoiding crowded places [11–13]. Individual avoidance behaviors that limit contact with others are forms of social distancing known as ‘informal social distancing’ [14]. Concerning to the behavioral response, we mainly focus on practicing recommended behaviors that is practicing hand hygiene and wearing facial masks, as well as social distancing that is reducing the use of public transport, avoiding crowded places, and postponing or canceling social events.

Risk Perception Attitude framework hypothesizes that an individual’s efficacy beliefs act as a key factor along with perceived risk in driving behavioral changes which is derived from the Extended Parallel Process Model [15]. Conceptually, efficacy beliefs comprise (a) beliefs about the effectiveness of the recommended response in discouraging, prevention of a health risk that is response efficacy or outcome expectations, and (b) an individuals’ perceived ability to exert personal control that is self-efficacy [16].

Ethiopia became a Covid-19 affected country on March 13, 2020. The government of Ethiopia took several progressive measures to combat the Covid-19 pandemic. Commonly known recommended preventive behavioral messages of COVID-19 in Jimma University Medical Center were properly washing hands with soap and water (95.5%), not touching eye-nose-mouth with unwashed hands (92.7%), and avoiding crowded places (90.3%) [17]. Furthermore, COVID-19 recommended preventive behavioral messages in Addis Ababa residents were 85% and 83% hand washing and social distancing respectively [18]. Despite all these important public health containment measures, the outbreak still has the potential for greater loss of life in Ethiopia if the community is unable to shape the regular behavioral and sociocultural norms that would facilitate the spread of the disease [19]. Studies were conducted in Knowledge, Attitude and Practice (KAP) on COVID-19, little is known in Ethiopia regarding response to the recommended preventive behavioral messages using the constructs of the Extended Parallel Process Model. Therefore, the aim of this study was to assess the response to COVID-19 recommended preventive behavioral messages based on this model.

## Methods and materials

### Study design and setting

This community-based cross-sectional study design was carried out from 1 to 20 July 2020 in Guraghe zone town administrative communities. The zone's capital city is Wolkite town, which is 158 km far from South of Addis Ababa. It comprises five town administrations; which are Wolkite, Butajira, Gunchire, Bu’i and Emdibir towns. They comprise 23 kebeles which are Wolkite have 7 kebeles, Gunchire have 4 kebeles, Butajira have 6 kebeles, Emdibir have 2 kebeles and Bu’i have 4 kebeles in total with a different number of population size.

### Study population

All adults of Guraghe zone town administrations; whose age greater than or equal to 18 years old were our source population. The study population was all sampled individuals living in the selected kebeles during the data collection period. The inclusion criteria were all selected individuals above 18 years old, and permanent residents of the selected kebeles. The Exclusion criteria were individuals in the selected kebele, who are patients with mental disorder unable to communicate properly, acute sick-looking persons who are unable to communicate during data collection, and under 18 years old.

### Sample size and sampling procedure

The sample size was calculated using single population proportion formula (n =  (Zα/2)2[ (PQ)/ (w)2]) with 95% confidence interval, 5% margin of error and proportion of response to Covid-19 recommended preventive behavioral messages of 50% since there was no previous study done in the area.

The design effect was determined to be 1.5 for having two stages in selecting study subjects. Hereafter, the calculation using Epi info version 3.5.3 yielded 576. The tolerable non-response rate is 10%. The final sample size became 634. Multi-stage systematic sampling technique was used to select study participants. In the first stage, we randomly select three town administrations namely Gunchire, Butajira and Wolkite of the Guraghe zone from a total of five. Next, we select respective kebeles that is 2 kebeles from Gunchire town, 3 kebeles from Butajira town and 4 kebeles from Wolkite town using a simple random sampling procedure. The final sample size was allocated proportionally to their total households in respective kebeles. “Kebele” is the lowermost administrative body in Ethiopia which includes at least 1000 households or population of 5000 people.

Then a systematic sampling technique was applied with sampling interval of every 8^th^ household from selected town kebeles. We interviewed the most decision-makers in the household either father or mother. If father or mother was not avail during data collection time, one eligible respondent was selected based on the lottery method from more than one eligible group. If eligible respondents are not found in the selected household, the immediate next house was visited. Closed house or unavailable respondents at the time of data collection continent time was rearranged to revisit them again without reluctance until found the respondent.

### Data collection procedure and data quality control

The data collection tools were semi-structured questionnaires, which are adapted from WHO and similar literature conducting on COVID-19 [15, 20, 21]. It was initially prepared in English then translated into Amharic by those proficient in the language and checked for consistency. Data collection was conducted using a face-to-face interview with selected households through applying the recommended preventive behavioral messages.

Before the main data collection was started pre-testing of the instrument on 5% (32 household) of the respondents conducted in Werabe town for clarity and flow of the questionnaire. Based on the finding appropriate correction was taken (including estimation of the time needed for data collection and those questions found to be unclear or confusing was modified). Reliability was checked using Cronbach alpha. From all reliability scores, the minimum reliability score was perceived efficacy which was 0.72.

Six data collectors who have health professional background was recruited and trained by an investigator with an assistant for two days. Two BSc Public Health professional were recruited as supervisors to monitor the progress of data collection and maintaining data quality. The supervisors were oriented on how to solve problems and ambiguities on the questions. The principal investigators were communicated with supervisors daily and followed the data collection closely. The collected data were checked for completeness and consistency.

### Study variables and operational definition

The dependent variable was response to COVID-19 recommended Preventive Behavioral Messages. The independent variables were sociodemographic and economic factors, knowledge, perceived susceptibility, perceived severity, self-efficacy, response efficacy, cues to action, message exposure, and recall.

#### Response to recommended preventive behavioral messages

Involves eight items of preventive behavioral messages were computed. Responses were recorded with Yes/No form. Individual scores for each preventive action were summed up and a composite score of response to message was created. Finally, the response was dichotomized based on the mean response score of the respondents. Those scored above the mean was considered as having good response and the rest as having poor response to COVID-19 recommended preventive behavioral messages.

#### Knowledge about COVID-19

These tools were adapted from WHO resources, which encompasses 14 questions. All the questions elicited a “yes/no” answer. Overall, three labels of knowledge status were created based on the number of correct responses that are; low (< = 8 of 14), moderate (9–10 of 14), and high (> = 11 of 14 items [22].

#### Perceived severity

It was measured through three items adapted from the Risk Behavior Diagnosis (RBD) scale using a five-point Likert scale. The scales are range from 1 to 5 that is from strongly agree to strongly disagree. The response was summed up and the total score was computed with possible values ranging from 3 to 15 then the score was treated as a continuous variable.

#### Perceived susceptibility

It was measured through three items adapted from the Risk Behavior Diagnosis scale using a five-point Likert scale. The scales are range from 1 to 5 that is from strongly agree to strongly disagree. The response was summed up and the total score was computed with possible values ranging from 3 to 15 then the score was treated as a continuous variable.

#### Perceived self-efficacy

It was measured using four items adapted from the Risk Behavior Diagnosis scale using a five-point Likert scale. The scales are range from 1 to 5 that is from strongly agree to strongly disagree. The response was summed up then the total score was computed with possible values ranging from 4 to 20. The score was treated as a continuous variable.

#### Perceived response-efficacy

It was measured using four items adapted from the Risk Behavior Diagnosis scale using a five-point Likert scale. The scales are range from 1 to 5 that is from strongly agree to strongly disagree. The response was summed up and the total score was computed with possible values ranging from 4 to 20 then the score was treated as a continuous variable.

#### Message exposure and recall

This tool adapted from previously published research on HIV is contextualized to fit into this specific study [15]. It was measured using six items including a preferred source of information, preferred channels, and frequently heard the message, the specific message heard of /seen, preferred message appeals.

#### Cues to action

This tool adapted from previously published research on breast self-examination is contextualized to fit into this specific study [21]. It was measured using six items with yes or no responses. The score was summed up and treated as a continuous variable.

#### Perceived threat

The score of perceived susceptibility and perceived severity was summed up to form a score of perceived threat.

#### Perceived efficacy

The score of perceived self-efficacy and perceived response efficacy was summed up to form a score of perceived efficacies.

### Data processing and analysis

The completed data were checked by the principal investigator in daily basis for completeness and consistency, then coded, and entered into Epi data 3.3.1 software and exported to SPSS version 23 statistical software for data analysis. Descriptive statistics were used to describe the study variables and presented using text, frequency, and proportions, and mean with standard deviation. In addition, variables with dichotomous outcomes were assessed using chi-square statistics. To identify factors associated with response to COVID-19 recommended preventive behavioral messages, a logistic regression model was used.

The bivariable logistic regression model was used for each explanatory variable to identify candidate variables with p-value < 0.25 for multivariable logistic regression. Adjusted odds ratio (AOR) with 95% CI was estimated to identify the associated factors. Finally, statistical significance was declared at p-value < 0.05 in multivariable logistic regression. Test for model fitness was done by using the Hosmer–Lemeshow model test. Hosmer and Lemeshow’s goodness-of-fit test was chi-square of 12.94 with a p-value of 0.11. Multi collinearity of the independent variables was checked by a variance inflation factor (VIF).

## Results

### Sociodemographic and economic characteristics of the participants

Six hundred thirty-three respondents were approached in this study, which indicated the response rate of 99.8%. The mean ± Standard Deviation age of the respondents was 34 ± 12.34 years. More than half of 325 (51.3%) were male respondents. More than two-fifth 278 (43.9%) of respondents were Muslim religion followers, 389 (61.5%) were married and 247 (39%) were joined higher education. The majority of the respondents; 322 (69.1%) were government employees in their occupation. The median (Interquartile Range) of the respondents’ monthly income was 2000 (3200) Ethiopian Birr (Table 1).

**Table 1:** Socio-demographic and economic characteristics of response to COVID-19 Preventive Behavioral Messages among Gurage Zone town administration communities, South Ethiopia, July 2020 (n = 633)

Variables	Response/categories	Frequency (n)	Percentage (%)
Age (years)	< = 29	283	44.7
	30–39	181	28.6
	40–49	88	13.9
	> = 50	81	12.8
Sex	Male	325	51.3
	Female	308	48.7
Marital status	Single	244	38.5
	Married	389	61.5
Religion	Muslim	278	43.9
	Orthodox	220	34.8
	Protestant	92	14.5
	Catholic	43	6.8
Education	Cannot read and write	37	5.8
	Read and write	124	19.6
	Primary education	90	14.2
	Secondary education	135	21.3
	Higher education	247	39.0
Occupation	Merchant	141	22.3
	Government employed	222	35.1
	Farmer	48	7.6
	Daily laborer	48	7.6
	Housewife	79	12.5
	Student	95	15.0
Monthly income (Ethiopian Birr)	< = 499	117	18.5
	500–1499	165	26.1
	1500–2999	100	15.8
	3000–4999	174	27.5
	> = 5000	77	12.2

### Preventive behavioral messages exposure to COVID-19

All of the respondents had exposed to COVID-19 preventive behavioral messages in the last six months. Television screen was the major channels to receive the information 570 (90.0%) and also preferred channel to see or hear message by respondents 573 (90.5%). From frequently heard of/seen message components, 626 (98.9%) respondents were exposed to properly washing hands with soap and water followed by not touching eye-nose-mouth with unwashed hands 587 (92.7%). Factual through education was preferred a message appeal among 447 (70.6%) respondents (Table 2).

**Table 2:** Preventive behavioral messages exposure and recall to COVID-19 among Gurage zone town administration communities, South Ethiopia July 2020 (n = 633)

Questions	Categories	Response	
		Yes (%)	No (%)
From where you received the information about COVID-19 preventive behavioral messages in the last 6 months	Health institutions	435 (68.7%)	198 (31.3%)
	Radio	447 (70.6%)	186 (29.4%)
	Television	570 (90.0%)	63 (10.0%)
	Parents/spouse	249 (39.3%)	384 (60.7%)
	Friends	311 (49.1%)	322 (50.9%)
	Religious institutions	400 (63.2%)	233 (36.8%)
	Posters	144 (22.7%)	489 (77.3%)
	Leaflets/brochures	23 (3.6%)	610 (96.4%)
	Social media	63 (10.0%)	570 (90.0%)
Preferred channels to hear/see	Television	573 (90.5%)	60 (9.5%)
	Radio	421 (66.5%)	212 (33.5%)
	Peer discussions	274 (43.3%)	359 (56.7%)
	Posters	155 (24.5%)	478 (75.5%)
	Leaflets/brochures	34 (5.4%)	599 (94.6%)
	Social media	68 (10.7%)	565 (89.3%)
Specific messages frequently heard of/seen	Properly washing hands with soap and water for a minimum of 30 s	626 (98.9%)	7 (1.1%)
	Not touching eye-nose-mouth with unwashed hands	587 (92.7%)	46 (7.3%)
	Avoiding walking to crowded places without face mask/social distancing	549 (86.7%)	84 (13.3%)
	Staying at home except for necessary issues	431 (68.1%)	202 (31.9%)
Preferred message appeals	Dramatic/funny	303 (47.9%)	330 (52.1%)
	Factual through education	447 (70.6%)	186 (29.4%)
	Fear arousal messages	108 (17.1%)	525 (82.9%)
	Two-sided messages	351 (55.5%)	282 (44.5%)
	One sided message	35 (5.5%)	598 (94.5%)
	Positive message	82 (13.0%)	551 (87.0%)
	Negative message	37 (5.8%)	596 (94.2%)

### Knowledge about COVID-19

To measure knowledge on the COVID-19 virus, 14 items were presented. The mean ± Standard Deviation score was 12.91 ± 1.36 for the respondents with, a range score of 7–14. The general correct answer rate of the knowledge questionnaire was 92.21% (12.91/14*100) while the range of correct answer rates for all respondents were between 50 to 100% (Table 3).

**Table 3:** Knowledge of COVID-19 among Gurage zone town administration communities, South Ethiopia, July 2020 (n = 633)

Knowledge of symptoms	Frequency (Percentage)	
	Correct	Incorrect
The main clinical symptoms of COVID-19 are fever, fatigue, dry cough, and myalgia	622 (98.3%)	11 (1.7%)
Unlike the common cold, stuffy nose, runny nose, and sneezing are less common in persons infected with the COVID-19 virus	571 (90.2%)	62 (9.8%)
Knowledge of high risk and prognosis		
Not all persons with COVID-19 will develop severe cases. Only those who are elderly, have chronic illnesses and obese are more likely to be severe cases	599 (94.6%)	34 (5.4%)
There currently is no effective cure for COVID-19, but early symptomatic and supportive treatment can help most patients recover from the infection	623 (98.4%)	10 (1.6%)
**Knowledge about Mode of transmissions and infectiousness**		
The COVID-19 virus spreads via respiratory droplets of infected individuals	595 (94.0%)	38 (6.0%)
Eating or contacting wild animals would result in the infection by the COVID-19 virus	491 (77.6%)	142 (22.4%)
Persons with COVID-19 cannot infect the virus to others when a fever is not present*	165 (26.1%)	468 (73.9%)
**Knowledge about ways of prevention**		
Proper washing hand with soap and water is one method of preventing COVID-19	630 (99.5%)	3 (0.5%)
One way of prevention of COVID-19 is not touching the eye, nose by unwashed hands	627 (99.1%)	6 (0.9%)
To prevent the infection by COVID-19, the individuals should avoid going to crowded places such as train stations and avoid taking public transportations	617 (97.5%)	16 (2.5%)
People who have contact with someone infected with the COVID-19 virus should be immediately isolated in a proper place	610 (96.4%)	23 (3.6%)
Ordinary residents can wear general medical masks to prevent the infection by the COVID-19 virus	592 (93.5%)	41 (6.5%)
Isolation and treatment of people who are infected with the COVID- 19 virus are effective ways to reduce the spread of the virus	546 (86.3%)	87 (13.7%)
Children and young adults do not need to take measures to prevent the infection by the COVID-19 virus*	52 (8.2%)	581 (91.8%)

*Correction rate calculated from ‘no’ response for false statements

### Multi-dimensional knowledge status of COVID-19

**Fig. 1:**
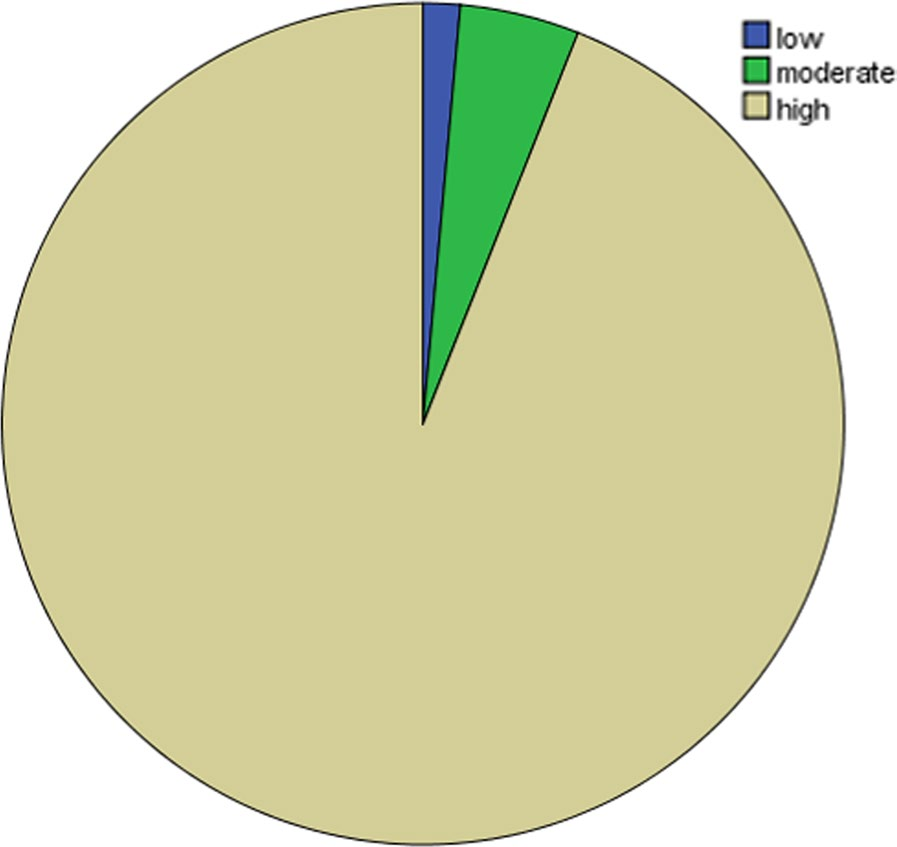
Pie charts show multi-dimensional knowledge status about COVID-19 among Guraghe zone town administration communities, South Ethiopia July 2020

Multidimensional knowledge (MDK) status encompasses symptoms, risk factors and prognosis, transmission modes, and preventive methods analysis of knowledge of COVID-19 indicated that 1.4%, 4.6%, and 94% of the communities were low, moderately, and highly knowledgeable respectively (*Fig.* *1**).*

### Risk perception and efficacy of the respondents

A detailed description of perception items intended to response to COVID-19 recommended preventive behavioral messages among Gurage zone town administration communities, South Ethiopia, July 2020 (Table 4).

**Table 4:** Perceived threat of COVID-19 among Gurage zone town administration communities, South Ethiopia, July 2020 (n = 633)

Questions	Response Categories				
Perceived susceptibility to COVID-19	Strongly Disagree	Disagree	Undecided	Agree	Strongly Agree
It is likely that I will get COVID-19	20 (3.2%)	170 (26.9%)	32 (5.1%)	374 (59.1)	37 (5.8%)
I am at risk for getting COVID-19	42 (6.6%)	193 (30.5%)	70 (11.1%)	302 (47.7%)	26 (4.1%)
It is possible that I will get COVID-19	15 (2.4%)	105 (16.6%)	33 (5.2%)	427 (67.5%)	53 (8.4%)
**Perceived severity of COVID-19**					
I believe that COVID-19 is severe	3 (0.5%)	26 (4.1%)	6 (0.9%)	320 (50.6%)	278 (43.9%)
I believe that COVID-19 has serious negative consequences	3 (0.5%)	23 (3.6%)	8 (1.3%)	330 (52.1%)	269 (42.5%)
I believe that COVID-19 is extremely harmful	2 (0.3%)	37 (5.8%)	11 (1.7%)	322 (50.9%)	261 (41.2%)
**Perceived response efficacy for Covid-19 preventive behavioral messages**					
Properly washing hands with soap and water could prevent risk of COVID-19	0	7 (1.1%)	298 (47.1%)	328 (51.8%)	0
Not touching eye-nose-mouth with unwashed hands could prevent risk of COVID-19	2 (0.3%)	10 (1.6%)	5 (0.8%)	303 (47.9%)	313 (49.4%)
Avoiding walking to crowded places without face mask could prevent risk of COVID-19	0	14 (2.2%)	2 (0.3%)	319 (50.4%)	298 (47.1%)
Staying at home could prevent risk of COVID-19	5 (0.8%)	25 (3.9%)	18 (2.8%)	348 (55%)	237 (37.4%)
**Perceived Self-efficacy to perform Covid-19 preventive behavioral messages **					
It is easy for me to engage on proper hand washing with soap and water which prevent risk of COVID-19	1 (0.2%)	40 (6.3%)	29 (4.6%)	397 (62.7%)	166 (26.2%)
I am able to adapt for not touching eye-nose-mouth with unwashed hands which prevent risk of COVID-19	8 (1.3%)	65 (10.3%)	71 (11.2%)	400 (63.2%)	89 (14.1%)
I am confident enough for avoiding crowded places could prevent risk of COVID-19	52 (8.2%)	185 (29.2%)	149 (23.5%)	214 (33.8%)	33 (5.2%)
Staying at home is easy for me which could prevent risk of COVID-19	258 (40.8%)	203 (32.1%)	79 (12.5%)	84 (13.3%)	9 (1.4%)

### Mean, Standard Deviation and reliability Scores of constructs of EPPM

Regarding risk perceptions, respondents had a Perceived threat mean score of 23.05 (± 3.870) and a perceived efficacy mean score of 30.49 (± 3.630). Cronbach’s α score for all the constructs was greater than 0.7 (Table 5).

**Table 5:** Respondents mean, standard deviation and reliability scores of constructs of the Extended Parallel Process Model among Gurage Zone town administration communities, South Ethiopia, July 2020 (n = 633)

Variables	Number of items	Response Range	Respondent’s Range	Mean (± SD)	Cronbach’s α
Perceived threat	6	0–30	6–30	23.05 (± 3.87)	0.81
Perceived susceptibility	3	0–15	3–15	10.13 (± 2.71)	0.85
Perceived severity	3	0–15	3–15	12.93 (± 2.07)	0.91
Perceived efficacy	8	0–40	17–40	30.49 (± 3.63)	0.72
Perceived response efficacy	4	0–20	8–20	17.61 (± 2.20)	0.87
Perceived self-efficacy	4	0–20	4–20	12.88 (± 2.83)	0.72

### Response to COVID-19 recommended preventive behavioral messages and risk perception (Perceived Threat and Perceived Efficacy)

From 425 respondents who had lower perceived threat, 194 (45.6%) had good response to preventive behavioral messages and the rest had poor. Regarding perceived efficacy from 324 respondents who were contained in low perceived efficacy category 136 (42%) had good response to preventive behavioral messages while the rest had poor response (Table 6).

**Table 6:** Shows chi-square association between response to COVID-19 disease preventive behavioral messages with risk perceptions among Gurage zone town administration communities, South Ethiopia, July 2020 (n = 633)

Perceived threat- efficacy interaction	Categories	Response to preventive behavioral messages/practice	
		Poor (%)	Good (%)
Perceived threat	Low threat	231 (54.4)	194 (45.6)
	High threat	105 (50.5)	103 (49.5)
Perceived efficacy	Low efficacy	188 (58.0)	136 (42.0)
	High efficacy	148 (47.9)	161 (52.1)

### Cues to action to prevent Covid-19 disease

To measure cues to action variable score to prevent COVID-19, six questions were raised. The mean ± Standard Deviation score was 8.04 ± 1.02 for the respondents with, a range score of 6–12 (Table 7).

**Table 7:** Cues to action to prevent COVID-19 disease among Gurage zone town administration communities, South Ethiopia, July 2020 (n = 633)

Items	Frequency (Percentage)	
	Yes	No
Have you ever seen /heard about a person who follow recommended preventive behavioral messages for COVID-19 in last one month?	591 (93.4%)	42 (6.6%)
Have you ever seen /heard of person who exposed to COVID-19 in the last one month?	546 (86.3%)	87 (13.7%)
Have you ever heard through mass media about recommended preventive behavioral messages for COVID-19 during last one month?	608 (96.1%)	25 (3.9%)
Have you ever heard through Health care providers about the recommended preventive behavioral messages for COVID-19 during last one month?	495 (78.2%)	138 (21.8%)
Have you ever heard through security workers about the recommended preventive behavioral messages for COVID-19 during last one month?	262 (41.4%)	371 (58.6%)
Do you have a family member who exposed to COVID-19?	2 (0.3%)	631 (99.7%)

### Response to COVID-19 recommended preventive behavioral messages

Regarding the measure of recommended preventive behavioral messages for COVID-19, eight items were presented. The mean ± Standard Deviation score was 4.38 ± 2.43 for the respondents with, a range score of 0–8. Predominantly undertaken behavioral items which prevent the risk of COVID-19, were engagement on proper handwashing with soap and water, used cover/elbow during coughing/sneezing, avoided walking to crowded places without wearing face mask, and usage of alcohol/sanitizer for rubbing and no contact with surfaces. While 366 (53.1%) of respondents had good response to COVID-19 recommended preventive behavioral messages, around 46.9% of respondents scored below the mean makes them poor response category (Table 8).

**Table 8:** Response to COVID-19 recommended preventive behavioral messages for the last consecutive days among Gurage zone town administration communities, South Ethiopia, July 2020 (n = 633)

Behavioral variables	Frequency (Percentage)	
Of the last consecutive few-weeks did you…	Yes (%)	No (%)
Engage on proper hand washing with soap and water, which prevent risk of COVID-19	563 (88.9%)	70 (11.1%)
Stopped shaking hands while giving greeting	304 (48%)	329 (52%)
Avoided proximity including while greeting (at least two-foot jump distance in between)	175 (27.6%)	458 (72.4%)
Practiced not touched eye-nose-mouth with unwashed hands	304 (48%)	329 (52%)
Avoided walking to crowded places without wore face mask	402 (63.5%)	231 (36.5%)
Practiced staying at home except for necessary issues	175 (27.6%)	458 (72.4%)
Used cover /elbow during coughing/sneezing	454 (71.7%)	179 (28.3%)
Used alcohol/sanitizer for rubbing and no contact with surfaces	397 (62.7%)	236 (37.3%)
**Response to recommended Preventive Behavioral messages (8 items)**		
Good response	336 (53.1%)	
Poor response	297 (46.9%)	

### Factors associated with response to COVID-19 recommended preventive behavioral messages

On bivariable logistic regression analysis, variables candidate for multivariable analysis were obtained from sociodemographic and economic factors; age, religion, education, occupation, and income. Knowledge and constructs of EPPM were also candidate for multivariable analysis (Table 9). On multivariable logistic regression analysis, merchant was 1.86 times more likely to respond to COVID-19 recommended preventive behavioral messages than government-employed [AOR = 1.86 (1.17, 2.96), p value = 0.01]. Respondents who scored one unit increase for self-efficacy and response-efficacy, the odds of responding to COVID-19 recommended preventive behavioral messages were increased by 1.22 and 1.05 times respectively. Respondents who scored one unit increase to cues to action, the odds of responding to COVID-19 recommended preventive behavioral messages were 43% less likely (Table 10).

**Table 9:** Bivariable logistic regression for response to COVID-19 recommended preventive behavioral messages among Gurage zone town administration communities, South Ethiopia, July 2020 (n = 633)

Variables	Categories	Response to COVID-19 recommended preventive behavior		COR (95% CI)	p-value
		Poor behavior	Good behavior		
Age	< = 29	141 (49.8%)	142 (50.2%)	1	0.15*
	30–39	98 (54.1%)	83 (45.9%)	0.84 (0.58,1.22)	0.36
	40–49	45 (51.1%)	43 (48.9%)	0.95 (0.59,1.53)	0.83
	> = 50	52 (64.2%)	29 (35.8%)	0.55 (0.33,0.92)	0.02
Sex	Male	170 (52.3%)	155 (47.7%)	1	
	Female	166 (53.9%)	142 (46.1%)	0.94 (0.69,1.28)	0.69
Marital status	Single	134 (54.9%)	110 (45.1%)	0.89 (0.64,1.22)	0.46
	Married	202 (51.9%)	187 (48.1%)	1	
Religion	Muslim	148 (53.2%)	130 (46.8%)	1	
	Orthodox	110 (50.0%)	110 (50.0%)	1.14 (0.80,1.62)	0.47
	Protestant	51 (55.4%)	41 (44.6%)	0.92 (0.57,1.47)	0.71
	Catholic	27 (62.8%)	16 (37.2%)	0.68 (0.35,1.31)	0.24*
Education	Cannot read and write	23 (62.2%)	14 (37.8%)	0.41 (0.20,0.83)	0.01*
	Read and write	71 (57.3%)	53 (42.7%)	0.50 (0.32,0.77)	0.00*
	Primary education	60 (66.7%)	30 (33.3%)	0.33 (0.20,0.56)	< 0.001*
	Secondary education	83 (61.5%)	52 (38.5%)	0.42 (0.27,0.64)	< 0.001*
	Higher education	99 (40.1%)	148 (59.9%)	1	
Occupation of the respondent	Government employed	92 (41.4%)	130 (58.6%)	1	
	Merchant	87 (61.7%)	54 (38.3%)	0.44 (0.29,0.68)	< 0.001*
	Farmer	34 (70.8%)	14 (29.2%)	0.29 (0.15,0.57)	< 0.001
	Daily laborer	31 (64.6%)	17 (35.4%)	0.39 (0.20,0.74)	0.004
	Housewife	49 (62.0%)	30 (38.0%)	0.43 (0.26,0.73)	0.002
	Student	43 (45.3%)	52 (54.7%)	0.86 (0.53,1.39)	0.53
Income	< = 499	73 (62.4%)	44 (37.6%)	0.59 (0.33,1.05)	0.07*
	500–1499	88 (53.3%)	77 (46.7%)	0.85 (0.50,1.47)	0.56
	1500–2999	54 (54.0%)	46 (46.0%)	0.83 (0.46,1.51)	0.54
	3000–4999	83 (47.7%)	91 (52.3%)	1.07 (0.63,1.83)	0.81
	> = 5000	38 (49.4%)	39 (50.6%)	1	
Source of information	β = 0.03			1.034 (0.95,1.12)	0.42
Knowledge	Poor	27 (71.1%)	11 (28.9%)	0.440 (0.21,0.90)	0.03*
	Good	309 (51.9%)	286 (48.1%)	1	
Perceived susceptibility	β = 0.34			1.11 (1.04,1.17)	0.10*
Perceived severity	β = 0.05			1.05 (0.97,1.14)	0.20*
Self-efficacy	β = 0.22			1.24 (1.17,1.32)	< 0.001*
Response efficacy	β = 0.04			1.01 (0.44,1.12)	0.11*
Cues to action	β = 0.67			0.51 (0.43,0.62)	< 0.001*

*COR* crude odds ratio, *CI* confidence interval

*Candidate variables for multiple regression which their p-value < 0.25 in the bivariable results

**Table 10:** Multivariable logistic regression analysis for response to COVID-19 recommended preventive behavioral messages among Gurage zone town administration communities, South Ethiopia, July 2020 (n = 633)

Variables	Categories	COR (95% CI)	AOR (95% CI)	p-value
Occupation of the respondent	Government employed		1	
	Merchant	0.44 (0.29,0.68)	1.86 (1.17, 2.96)	0.01*
	Farmer	0.29 (0.15,0.57)	0.53 (0.25, 1.14)	0.10
	Daily laborer	0.39 (0.20,0.74)	0.77 (0.37, 1.61)	0.48
	Housewife	0.43 (0.26,0.73)	1.14 (0.61, 2.14)	0.68
	Student	0.86 (0.53,1.39)	1.72 (0.98, 3.04)	0.06
Cues to action	β = 0.57	0.51 (0.43,0.62)	0.57 (0.47, 0.69)	< 0.001**
Self- efficacy	β = 0.20	1.24 (1.17,1.32)	1.22 (1.14, 1.30)	< 0.001**
Response-efficacy	β = 0.13	1.01 (0.44,1.12)	1.05 (1.03,1.09)	0.00*

Hosmer and Lemeshow’s goodness-of-fit test was chi square of 12.94 with P-value of 0.11

*AOR* Adjusted odds ratio

*Variables statistically significant at p-value < 0.05

**Variables statistically significant at p-value < 0.001

## Discussion

This study was assessed response to COVID-19 recommended preventive behavioral messages which is one of the main implications for minimizing the rapid spread of the coronavirus. The COVID-19 is coming from a relatively new virus that has had overwhelming effects within a short period meanwhile it was first detected in December 2019. Predominantly undertaken behavioral items, which could prevent the risk of COVID-19, were engagement on proper handwashing with soap and water, used cover/elbow during coughing/sneezing, avoided walking to crowded places without wearing face mask and usage of alcohol/sanitizer for rubbing and no contact with surfaces.

In general, in this study the behavioral messages variable score was encompassing majorly performed preventive methods which were classified as good and poor response to COVID-19 recommended preventive behavioral messages. Even though the respondents were highly knowledgeable regarding prevention methods for infection of COVID-19, 53.1% of the respondent’s score were above the mean or good response to COVID-19 recommended preventive behavioral messages. This implies that there is a minimum level of response to COVID-19 recommended preventive behavioral messages for this specific study area than Jimma University Medical Center visitors and Addis Ababa residents [17, 18].

This finding is lower than the study done in Jimma University Medical Center visitors; properly washing hands with soap and water (95.5%), not touching eye-nose-mouth with unwashed hands (92.7%), and avoiding crowded places (90.3%) were commonly known recommended preventive behavioral messages of COVID-19 [17]. Furthermore, the study done in Addis Ababa residents showed that some of the recommended preventive behavioral messages of COVID-19 were 85% and 83% hand washing and social distancing respectively [18]. To close the clear gap between what was known and practicing the recommended behavioral messages; different stakeholders initiating start from individual to the large public should be struggling to minimize this pandemic disease.

These discrepancies may be raised from the time when data collection was conducted. As a result of redundantly hearing and seen about COVID-19; there was ignorance and lack of commitment for applying the recommended preventive behavioral messages. In Jimma University Medical Center visitors and Addis Ababa residents’ data collection was conducted early in the pandemic, so that they should be seriously applied concerning to preventive practice. Moreover, Ethiopia is known for modest coverage and shortage of water supply and handwashing facilities, a high rate of overcrowded living conditions, frequent social and religious ceremonies, and a high unemployment rate calling for crucial efforts were reason for not to be sustain in coherent manner regarding response to recommended preventive behavioral messages.

The study showed that merchant was 1.86 times more likely to respond to COVID-19 recommended preventive behavioral messages than government-employed [AOR = 1.86 (1.17, 2.96), p value = 0.01]. In line with this study, a study done in Jimma University Medical Center visitors working for private and government businesses were positive predictors of avoidance of shaking hands for the greeting which is majorly applied preventive practice for COVID-19 [17]. In line with this study, a study done in Addis Ababa found that occupation was found to be associated with the practice of precautionary measures against COVID-19 [18]. This implies that mainly merchants are more cautious for these catastrophic diseases than government employer because most of the time they have close contact to different people so that follow strict instructions.

A one unit increase for the score of self-efficacies, the odds of responding to COVID-19 recommended preventive behavioral messages were increased by 1.22 times. In line with this study, a study done in Jimma University Medical Center visitors stated that visitors who felt self-efficacious to successfully control COVID-19 were more likely to avoid handshaking to combat COVID-19. It depicts that these people were more concerned about contacts and adapted to hygienic precautions [17]. To increase the magnitude of preventive practice, the study participants have a capacity on engaging in proper handwashing with soap and water, the ability to adapt to not touching eye-nose-mouth with unwashed hands, being confident enough for avoiding crowded places, and easy for me to stay home.

A one unit increase for the score of response efficacy, the odds of responding to COVID-19 recommended preventive behavioral messages were increased by 1.05. This implies that the score of response efficacy was mainly handled with performing the recommended preventive behavioral messages. So that to increase applying preventive behavioral messages, the communities should be strengthening through properly washing hands with soap and water, not touching eye-nose-mouth with unwashed hands, avoiding walking to crowded places without a face mask, and also staying at home.

Respondents who scored one unit increase to cues to action, the odds of responding to COVID-19 recommended preventive behavioral messages were 43% less likely. This might be redundantly hearing or seeing extreme fear arousal messages, and also gathering misinformation leads to ignorance of the messages (defensive avoidance) or just not response at all to recommended COVID-19 preventive behavioral messages [15]. To increase the level of response to recommended preventive behavioral messages, the communities should give emphasis to the recommended preventive behavioral messages and also strengthening through avoiding ignorance, misinformation and filling extreme fear for COVID-19 pandemic.

### Limitations of the study

Though this study was mainly emphasized on preventive behavioral messages, difficult to assess the respondents’ actual behavior during once upon a time that is during data collection period. Even though many variables scored as continuous one that considered us our strength, it was faced challenge to discuss with similar categorical variables. Furthermore, unique constructs of the model that was response efficacy and cues to action were difficult for discussion with other literature. We were incapable to directly compare the perceived risk and efficacy of COVID-19 for the same respondents. Therefore, it is difficult to compare the absolute values of the perceived risk and efficacy. Our study aimed to identify factors affecting response to COVID-19 recommended preventive behavioral messages, rather than testing theoretical hypothesis of Extended Parallel Process Model.

## Conclusion

Even though respondents were highly knowledgeable about Covid-19, there is a lower level (53.1%) of response to recommended preventive behavior messages for this catastrophic disease. Merchant, self-efficacy, response efficacy, and cues to action were significantly associated with response to recommended preventive behavioral messages. Like merchants, government employer should be applying preventive behavioral messages and also, participants’ self and response efficacy should be strengthened to improve the response. In addition, we should be changed or modified the way how-to deliver relevant information, promoting awareness, and also using appropriate reminder systems to preventive behavioral messages.

## Data Availability

All relevant data are within the manuscript and there is no supplementary information provided.

## Abbreviations

AOR: Adjusted odds ratio
CI: Confidence interval
COR: Crude odds ratio
EPPM: Extended Parallel Process Model
JUMC: Jimma University Medical Center
HH: House hold
RBD: Risk behavioral diagnosis
SARS: Systemic acute respiratory syndrome
SPSS: Statistical Package for Social Science
WHO: World Health Organization
